# Strain-enhanced high *Q*-factor GaN micro-electromechanical resonator

**DOI:** 10.1080/14686996.2020.1792257

**Published:** 2020-07-27

**Authors:** Liwen Sang, Meiyong Liao, Xuelin Yang, Huanying Sun, Jie Zhang, Masatomo Sumiya, Bo Shen

**Affiliations:** a International Center for Materials Nanoarchitectonics (MANA), National Institute for Materials Science (NIMS), Tsukuba, Ibaraki, Japan; b Amano-Koide Collaborative Research Lab, National Institute for Materials Science, Tsukuba, Ibaraki, Japan; c Wide Bandgap Materials Group, National Institute for Materials Science (NIMS), Ibaraki, Japan; d State Key Laboratory of Artificial Microstructure and Mesoscopic Physics, School of Physics, Peking University, Beijing, China

**Keywords:** GaN, MEMS resonator, stress, lattice mismatch, 201 Electronics / Semiconductor / TCOs

## Abstract

We report on a highly sensitive gallium nitride (GaN) micro-electromechanical (MEMS) resonator with a record quality factor (*Q*) exceeding 10^5^ at the high resonant frequency (*f*) of 911 kHz by the strain engineering for the GaN-on-Si structure. The *f* of the double-clamped GaN beam bridge is increased from 139 to 911 kHz when the tensile stress is increased to 640 MPa. Although it is usually regarded that the energy dissipation increases with increasing resonant frequency, an ultra-high *Q*-factor which is more than two orders of magnitude higher than those of the other reported GaN-based MEMS is obtained. The high *Q*-factor results from the large tensile stress which can be intentionally introduced and engineered in the GaN epitaxial layer by utilizing the lattice mismatch between GaN and Si, leading to the stored elastic energy and drastically decreasing the energy dissipation. The developed GaN MEMS is further demonstrated as a highly sensitive mass sensor with a resolution of 10^−12^ g/s through detecting the microdroplet evaporation process. This work provides an avenue to improve the *f × Q* product of the MEMS through an internally strained structure.

## GRAPHICAL ABSTRACT

**Figure:**
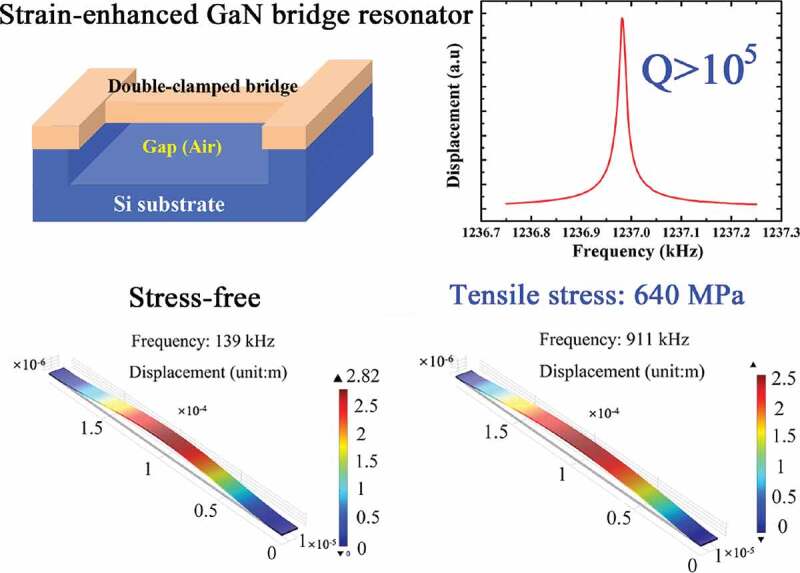

## 1. Introduction

Silicon-based nano- and micro-electromechanical systems (NEMS/MEMS) are reaching their limits for sensing in harsh conditions, a matter that has received extensive attentions in recent years. With the increasing requirement for MEMS/NEMS regarding novel functionalities, the wide-bandgap semiconductors are attracting more attentions due to their higher mechanical and thermal stability, biocompatibility, miniaturization, and facile integration [1–3]. Among all the wide-bandgap material systems, gallium nitride (GaN) intrinsically has a direct bandgap, superior mechanical properties, high thermal stability, and chemical inertness [4,5]. Especially, the high crystalline-quality GaN has been achieved on the silicon (Si) substrates in recent years. The GaN-on-Si technology enables the monolithic integration of MEMS/NEMS with the existing CMOS circuits, which is beyond GaN-on-sapphire or silicon carbide (SiC) technology [6]. The well-developed power electronic devices and high-frequency devices based on aluminium gallium nitride/gallium nitride (AlGaN/GaN) high electron mobility transistors (HEMTs) on Si substrates can be combined or integrated into MEMS to produce novel smart devices and systems [7–10]. However, compared to the well-developed optoelectronic devices, the GaN-based MEMS/NEMS technology is still in its infancy. For example, the reported quality (*Q*) factors of all the GaN or HEMT MEMS/NEMS systems are only on the order of 10^2^–10^3^, which are much lower than those of Si or SiC resonators [1,4,5].

For a mechanical resonator, the *Q*-factor and the resonance frequency are the two important parameters determining the sensitivity of the MEMS/NEMS [11–13]. For example, in the mass sensing, the addition of a small mass will change the resonator’s natural resonance frequency. Precise monitoring of the natural resonance frequency will detect a change of $\Delta \omega /\omega_{0}=-\text{\textbackslash{}Deltam}/2m$ due to an added mass \Deltam, so a system with a minimum frequency resolution can detect a minimum-added mass of $\Delta m_{min}=2m\Delta \omega_{min}/\omega_{0}$. Assuming that the mass adds uniformly to the mass of the overall resonator, if we take the simple rule that the minimum detectable change is that which gives a fractional change in frequency equal to the Allan variance, then [13]

(1) $$\frac{\delta m}{m}=\frac{1}{2}\frac{\delta \nu }{\nu }=\sqrt{\frac{k_{B}T}{k_{eff}L^{2}}\frac{1}{Q\omega \tau_{A}}}=\sqrt{\frac{k_{B}T}{mL^{2}}\frac{1}{Q\omega^{3}\tau_{A}}}$$

where ω=2πν is the natural resonance frequency of the resonator, *L* is the beam length, $\tau_{A}$ is the integration time, $k_{B}T$ is the thermal energy, and $k_{eff}$ is the effective spring constant. As can be seen, to improve the mass sensitivity, the increase of the *f* × *Q* product is required. In general, there is a trade-off between the resonant frequency and the *Q*-factor. For example, for the conventional cantilevers or bridges in which the clamping loss is the dominant energy dissipation mechanism, downscaling the thickness and increasing the length lead to the enhanced quality factor and reduced resonant frequency [14]. Therefore, it is highly desirable if both the *f* and *Q*-factor can be improved.

In this paper, we propose to simultaneously enhance the *Q*-factor and resonant frequency of the GaN MEMS by intentionally introducing an internal strain during the epitaxial growth. Through utilizing the lattice mismatch between Si and GaN, the tensile stress as large as 640 MPa is achieved in the double-clamped beam even after releasing Si. The high strain leads to the GaN resonator with a record-high *Q*-factor of more than 10^5^ and simultaneously a 6.6-fold increased resonant frequency compared to that of the unstrained one. The GaN double-clamped beam resonator is further demonstrated as an accurate mass sensor with a sensitivity of 10^−12^ g/s for the microdroplet evaporation process by the in-situ measurement of the resonant frequency shift in a vacuum chamber.

## 2. Methods, materials and devices

### 2.1. Methods to achieve high Q-factors for the NEMS/MEMS resonators

For NEMS/MEMS resonators, the *Q*-factors can be improved by a number of methods, such as the reduction of the material bulk/surface defects to reduce damping, lengthening the resonators, or external pumping to increase the stored energy [11,15]. Here, benefitting from the lattice mismatch between the GaN and Si, we intentionally introduce the stress in the GaN to help to store energy and improve the quality factors. The internal stress can be independently modulated by changing the growth conditions or the interlayers for the GaN grown on Si. This unique property provides an alternative tolerance for the strain engineering in the GaN-based NEMS/MEMS system. Moreover, different from the external tension, the internal strain is uniform and no external force is needed. Taking the pre-stress *σ* into consideration, the resonant frequency of the double-clamped beam resonators can be estimated by the Rayleigh method from the following equation [1,16,17],

(2) $$f_{n,\sigma }=1.028\sqrt{\frac{E}{\rho }}\frac{t}{L^{2}}\sqrt{1+0.295\frac{\sigma L^{2}}{Et^{2}}}$$

where *E* is the Young’s modulus, *ρ* the mass density, *t* the thickness, and *L* the length.

### 2.2. Strain control for the GaN epitaxially grown on Si

A high-quality GaN epitaxial layer is necessary for the NEMS/MEMS to reduce the localized defects-induced damping [18]. For the GaN grown on Si, the AlGaN or AlGaN/aluminium nitride (AlN) superlattices were typically used between AlN/Si buffer and GaN as the interlayers to block dislocations and avoid cracks. In this work, to introduce the strain in the epitaxial GaN, we developed a large lattice-mismatch induced stress control techniqueand directly deposit GaN on the AlN/Si (111) buffer without any strain-relief layers in the metal-organic chemical vapor deposition [19]. The Si substrate was initially treated with the exposure to the trimethyaluminium. Then, a 45 nm-thick low-temperature AlN nucleation layer was grown at 990°C, followed by a 225 nm-thick high-temperature AlN buffer layer grown at 1100°C. The following GaN epilayer with a thickness of 500 nm was thereafter deposited at the temperature of 1040°C (sample A). Although a large tensile stress was introduced, no cracks were observed on the GaN surface. For the reference, the GaN epilayers grown by the typical process with an AlGaN interlayer were prepared. The AlGaN was introduced on the AlN buffer with a thickness of 330 nm and an Al content of ~23%. The thickness of the GaN was 3 μm (sample B) and 2 μm (sample C in *Supplementary information*) at the same temperature of 1040°C.

### 2.3. Fabrication of the GaN MEMS resonators

The double-clamped GaN bridges with a width of 10 μm and different lengths from 100 to 400 μm were patterned by a laser lithography process. The inductively coupled plasma (ICP) etching was performed with Cl_2_/Ar mixed gases to etch the whole GaN/AlN for the MEMS structure. Finally, the resonators were released by wet-chemical etching the Si substrate with the HF:HNO_3_ (1:15) mixed solutions.

### 2.4. Materials and device characterization

The crystalline qualities of the GaN were studied by high-resolution transmission electron microscopy (HR-TEM, Tecnai F30) and X-ray diffraction (XRD, PANalytical) and scanning electron microscopy (SEM, FE-SEM (S-4800)). The out-of-plane resonant frequencies were detected by an optical interferometric velocity with a Doppler effect of a focused laser (He-Ne laser, 633 nm, <1 mW) at a vertical incidence to the substrate. The characterization was performed in an ultra-high vacuum chamber (~10^−4^ Pa) to eliminate the air damping loss at room temperature. The actuation of the resonators was performed by using a radio-frequency signal to drive a piezoceramic element that was physically placed at the bottom of the GaN samples. The frequency spectra were generated by Fast Fourier Transformation (FFT) of the velocity signals. The Raman scattering measurements were carried out by using an Ar-Kr green laser (514.5 nm) as the excitation light in a backscattering geometry with 1770 lines/mm grating, microscope, and charge-coupled device detector cooled by liquid nitrogen.

## 3. Results and discussion

### 3.1. Microstructure and strain analysis for the GaN epitaxial layers

**Figure 1.**
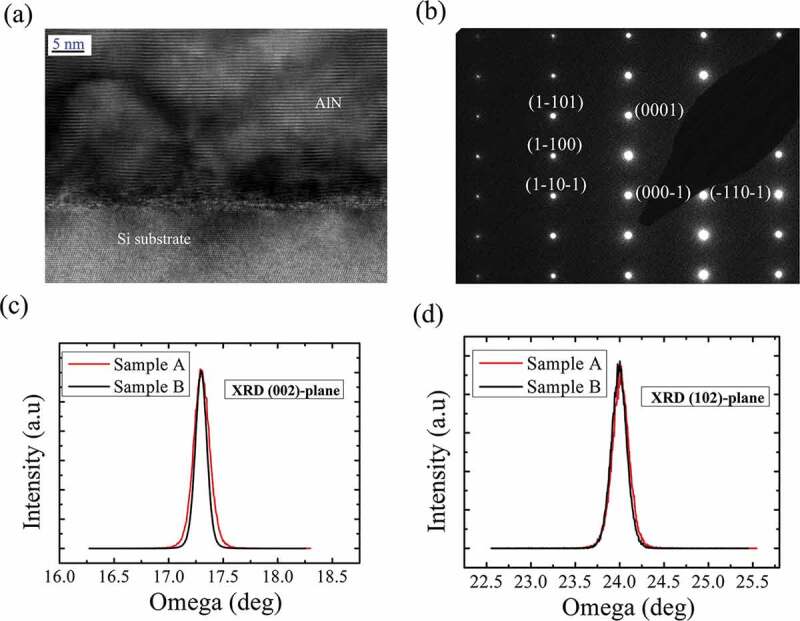
(a) HR-TEM image at the AlN/Si interface along [11–20]_AlN_/ [1–10]_Si_ direction. (b) The diffraction pattern of the high-quality single-crystalline GaN epilayer. (c) XRD rocking curve for the GaN (002)-plane. (d) XRD rocking curve for the GaN (102)-plane.

Figure 1(a,b) are the HR-TEM images of the AlN/Si interface along the [11–20]_AlN_/ [1–10]_Si_ direction and the diffraction pattern of GaN for sample A. As can be seen, the large tensile strain results in the lattice distortion of the AlN buffer and the interface misfit dislocations/domains. A large full-width at half maximum (FWHM) value of 960 arcsec around (002)-plane from XRD for AlN is observed. Since most of the misfit dislocations are controlled within the AlN buffer, the high-quality GaN epitaxial layer was obtained even when the thickness is as thin as 500 nm. The FWHMs of the XRD from the GaN (002) and (102)-planes are 550 and 716 arcsec, respectively, as illustrated in Figure 1(c,d). These values are comparable with the 3 μm-thick GaN (MEMS sample B, 444 arcsec for (002)-plane and 708 arcsec for (102)-plane), indicating a high crystalline quality of the nano-thick GaN on Si.

**Figure 2.**
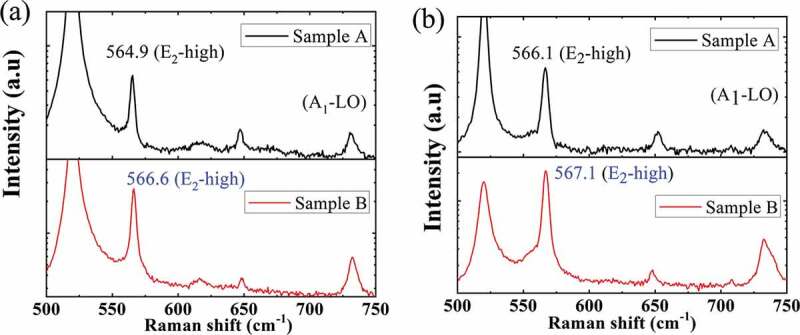
Raman spectra of sample A and B (a) before and (b) after release (for the bridge resonator with the length of 200 μm).

The internal stress in the GaN layer before and after releasing Si was analysed by the confocal micro-Raman scattering measurement. Figure 2(a,b) is the Raman spectra of sample A and B for the 200 μm-length beams. The peak at 519.3 cm^−1^ is from the Si substrate and is used as the calibration. The *E_2_*-high modes for GaN in the Raman spectra are utilized to examine the strain states as it has been proven particularly sensitive to the biaxial stress in III–V nitride semiconductors. As expected, the strain in all the GaN epilayers before release is tensile because the *E_2_*-high peaks exhibit red shifts with respect to the standard value of 568 cm^−1^ for the bulk GaN [20]. The shift of GaN *E_2_*-high peak in sample A is close to 2 cm^−1^, which is much higher than that of sample B, indicating a larger internal tensile stress. After removing the Si, the stresses are partially relaxed, but the clamping ends still induce tensile strain like a string. The *E_2_*-high peak of GaN in sample A displays a blue shift of 1.2 cm^−1^ before and after release, while the residual stress is still higher than in the thicker GaN beam. As shown in Figure 2(b), the *E_2_*-high peak of GaN in sample A is lower than that in sample B after releasing Si. It is noted that the AlN layer in sample A is nearly totally relaxed after release. A large shift of 5.1 cm^−1^ from 647.6 cm^−1^ for the strained epilayer to the unstrained value of 652.7 cm^−1^ is displayed. Unfortunately, the precise estimation of the tensile stress in the resonators is difficult since the value of the GaN Raman stress factor was scattered from −2.7 to −7.7 cm^−1^/GPa [21–25].

### 3.2. GaN MEMS resonant property and energy dissipation

**Figure 3.**
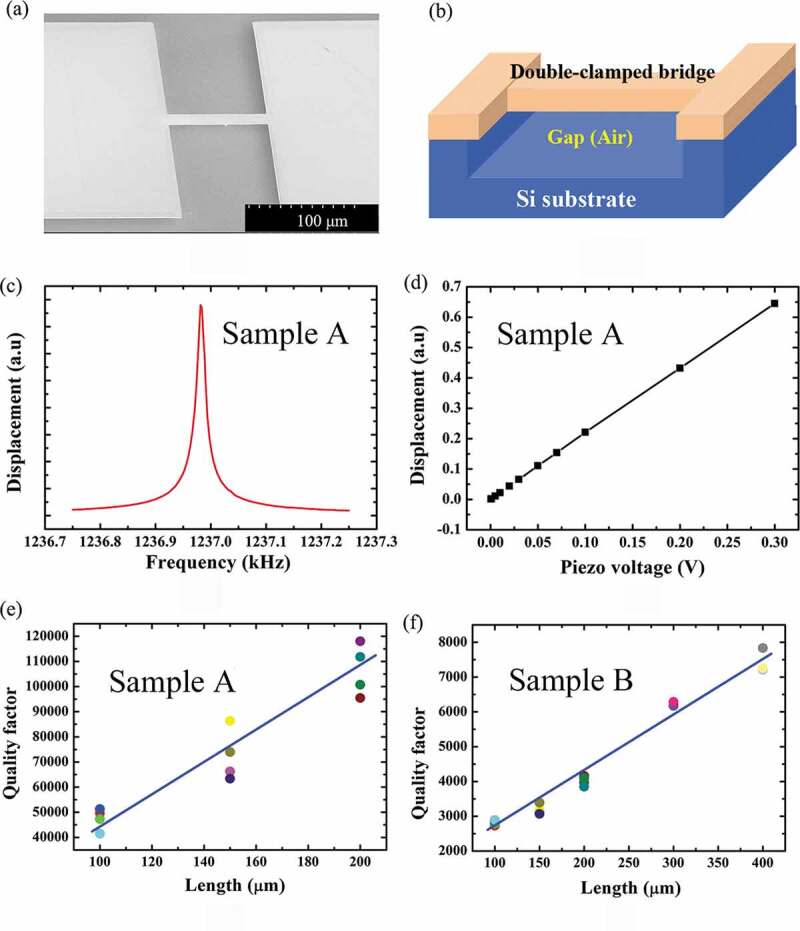
(a) A typical SEM image of the released double-clamped GaN bridge resonator. (b) Schematic of the bridge, showing the gap below the beam. (c) A typical optical resonant frequency spectrum of sample A in a width of 10 μm and length of 200 μm. (d) The displacement amplitude of the bridge dependent on the driving voltages. The quality factor *Q* of the double-clamped beams dependent on the length of the GaN beam bridges for (e) sample A and (f) B, respectively.

Figure 3(a) illustrates the typical scanning electron microscopy (SEM) image of the released double-clamped GaN bridge resonator. The schematic configuration of the bridge is presented in Figure 3(b), showing the gap below the bridge. The resonant frequency spectrum of sample A with a length of 200 μm is shown in Figure 3(c). The bridge behaves as a harmonic oscillator, the spectrum of which follows a square-root of Lorentzian profile with a centered frequency. During the measurements, the amplitude (*V_p_*) of the *ac* actuation signal applied to the piezoceramics was carefully controlled to ensure the symmetry of the resonant frequency spectra. The displacement amplitude of the bridge increases linearly with the driving voltage in the investigated range, as displayed in Figure 3(d), indicating a good resonant characteristic. The quality factors were extracted from the FWHMs of the resonant spectra by the Lorentzian fits. The *Q*-factor for sample A with the length of 200 μm was estimated to be 118,056, which is 2 ~ 3 orders of magnitude higher than those previously reported in the GaN/Si or HEMT/Si systems [5,17]. On the other hand, for sample B and sample C with thicker GaN epilayers in the same geometry, the *Q* values are much lower. Figure 3(e,f) compares the double-clamped beams dependent on the length of the GaN resonators for samples A and B, respectively. As can be seen, the *Q*-factors of sample A are in the range of 90,000–120,000 for the 200 μm-length beam, while they reduce to 3500–4000 for sample B in the same length. For all the samples, the *Q*-factors increase with increasing beam length.

To analyze the energy dissipation in the NEMS/MEMS resonators, several mechanisms are considered for the *Q*-factors, which include the air damping, support loss, thermoelastic damping (TED), and bulk or surface effect. As our measurement was performed in vacuum, the air damping can be neglected. The *Q*-factor deduced from the TED is theoretically more than 6 × 10^6^ at room temperature [26]. Therefore, the TED is not the dominant mechanism although its effect becomes important at high temperatures.

Then, we consider the clamping loss. For a flexural resonator with a finite base, the *Q*-factor due to the clamping loss can be expressed as Photiadis and Judge model [14,27],

(3) $$Q_{clamping}=\beta \frac{L}{w}{\left(\frac{t_{b}}{t}\right)}^{2}$$

where *β* is a constant, *w* the resonator width, and *t_b_* the basement thickness. As shown in Figure 3(e,f), the *Q*-factors for the bridges with different thicknesses show a nearly linear dependence on the resonator length, which seems to follow the above equation. The clamping loss based on this model displays an inverse power law (*1/t^2^*) dependence on the resonator thickness which can explain the case of samples B and C. However, the predicted *Q*-factors (75,000–80,000) for sample A are lower than the experimental ones (>10^5^). Since the only difference among the three samples is the residual stress inside the double-clamped beams, the higher *Q*-factors obtained from sample A are attributed to the much larger residual tensile stress than the thicker ones. As the *Q*-factor’s definition, it is the ratio of the stored energy versus lost energy over one cycle of the oscillation. While in the bridge or the membrane structures, the resonators can additionally store and dissipate energy in the lateral elongation [28–30]. Then, the quality factor of the double-clamped beam for the GaN resonators can be described by [30]:

(4) $$Q=2\pi W_{tension}+W_{elongation}+W_{bending}/\Delta W_{elongation}+\Delta W_{bending}$$

where *W_tension_* is the elastic energy stored in the resonator, Δ *W_bending_* and *W_bending_* are the lost and stored energy, respectively, due to the bending. It is obvious that the *Q* is enhanced by the stored tensile energy. Assuming that the magnitude of the tensile prestress dominates the mechanical behavior, the energy stored in the flexural bending can be neglected and the quality factor can be written as:

(5) $$Q\approx Q_{bending}*\frac{W_{tension}}{W_{bending}}\approx Q_{bending}*\frac{\frac{1}{2}\sigma A\int_{0}^{L}{\left[\frac{\partial }{\partial x}u\left(x\right)\right]}^{2}dx}{\frac{1}{2}EI_{Z}\int_{0}^{L}{\left[{\frac{\partial }{\partial x}}^{2}u\left(x\right)\right]}^{2}dx}$$

where *σ* is the tensile stress, *A* is the cross-section area, and *L* is the length, *E* is the Young’s modulus, *u(x)* is the bending mode shape and the *Q _bending_* is the quality factor due to bending related damping mechanisms in the relaxed state. As can be seen, the quality factor can be increasing when there is pre-stress. It should be pointed out that, since the AlN buffer layer is nearly totally relaxed by Raman measurement, the large tensile strain is considered to be mainly coming from the GaN epitaxial layer.

**Figure 4.**
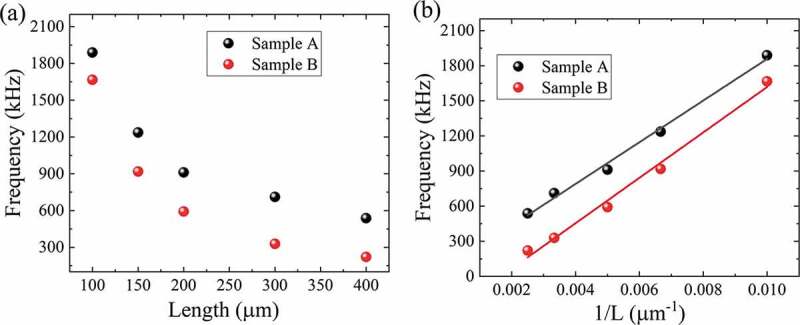
(a) Characteristics of the resonant frequencies *vs* bridge length for the resonators from sample A and B. (b) The resonant frequencies of all the three samples scale well with *1/L.*

The residual tensile stress can be accurately obtained by the resonant frequency shift of the mechanical resonators, which is much more precise than the micro-Raman analysis. Figure 4(a,b) shows the characteristics of the resonant frequencies *vs* bridge lengths for samples A and B. As can be seen, the *f* of all the samples scale well with *1/L*, rather than the typical *1/L^2^* [14]. Therefore, the strain-related term dominates the energy dissipation for the GaN bridge resonators. According to Equation (2), the residual stress can be determined. For sample A, the extracted stress is tensile with a value of 640 MPa for the beam in a length of 200 μm. For sample B in the same length, the tensile stress is 203 MPa. The tensile stress for sample C is 330 MPa, as shown in Fig. S1 in the *Supplementary information*. Therefore, we believe the internal strain leads to the marked enhancement of the *Q*-factor. The resolution of the estimated residual stress by resonant frequency measurement is much more precise compared to Micro-Raman spectra. It should also be pointed out that as the beams are released by the chemical etching, the anchor loss from the overhangs at the bridge edges is unavoidable. From the above discussion, the resonance frequency for a strained double-clamped beam is proportional to *1/L*, and the *Q*-factor is proportional to *L*. The overhangs increase the length of the beam, which leads to a slight reduction of the resonance frequency and increase of the *Q*-factor. Judging from the drastic increase of the resonance frequency, the effect from the Si overhangs is very small in the double-clamped beams.

### 3.3. NEMS/MEMS vibration mode simulation

**Figure 5.**
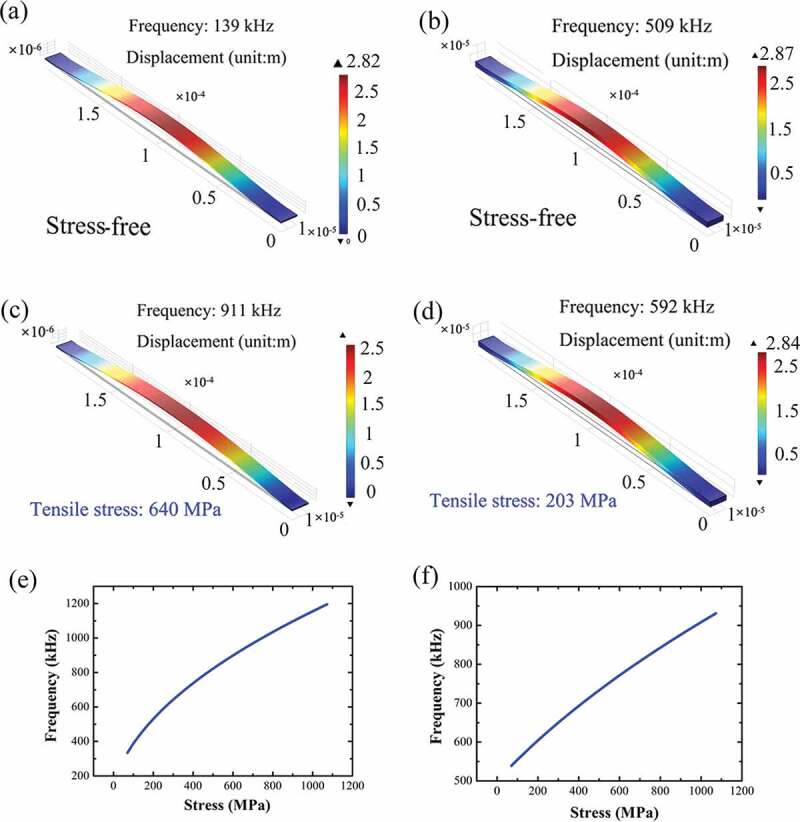
Finite element simulation for the resonant characteristics of (a) sample A and (b) sample B in the length of 200 μm without any stress. Finite element simulation for the resonant characteristics of (c) sample A under the tensile stress of 640 MPa and (d) sample B under the tensile stress of 203 MPa. The resonant frequency dependent on the stress is shown in (e) for sample A and (f) for sample B.

The resonant mode and the eigenfrequency of the GaN double-clamped beams are further analyzed by the finite element simulation, as shown in Figure 5. For sample A with the 500 nm-thick beam without the residual stress, the eigenfrequency is at 139 kHz for the fundamental bending mode along the *z* direction (Figure 5(a)), while it is enhanced drastically to 911 kHz (by 6.6 times) under the tensile stress of 640 MPa (Figure 5(c)), well consistent with the experimental value. The enhancement of the resonant frequency for the thicker resonator is reduced. A smaller enhancement from 509 kHz (Figure 5(b)) to 592 kHz (Figure 5(d)) is obtained for the 3 μm-thick resonator under the stress of 203 MPa. For the 2 μm-thick resonator, the resonant frequency is increased from 361 kHz to 709 kHz under the tensile stress of 330 MPa (Fig. S3 in the *Supplementary information*). The increases of *f* dependent on the stress for the resonators with different thickness are summarized in Figure 5(e,f) for samples A and B, respectively.

### 3.4. Highly sensitive mass sensors

Since the tensile stress improves both the quality factor and resonance frequency, while holding the resonator mass and length, the resolution of mass detections can be improved according to Equation (1). The MEMS-based mass sensor can be an ideal tool to measure the mass of micro- and nanosized particles and to characterize various physical processes. The sensor measures the shift in the resonant frequency of the structure before and after the attachment of the target entity, where the shift in the resonant frequency can be used to calculate the mass variation. In a range of physical and biological applications, the accurate estimation and precise control of the evaporation process of the microdroplets are very important. However, due to the lack of the appropriate measurement tools and the lack of corrosive resistance of the materials, the evaporation process has not been well characterized. Benefiting from the superior chemical stability of the wide-bandgap semiconductors, GaN MEMS resonators are promising for the detection of the various microdroplets. Here, we demonstrate a high resolution of 10^−12^ g with the developed GaN MEMS resonator for the uniform mass detection to directly evaluate the evaporation process of the methanol.

**Figure 6.**
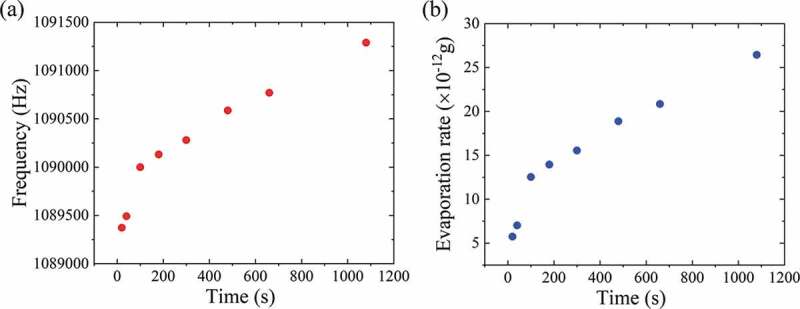
(a) The dependence of the resonant frequency shift with time for the methanol microdroplet evaporation. (b) The estimated evaporation rate for the methanol microdroplet in vacuum.

The methanol microdroplets are deposited using a microinjector, and the shift of resonant frequency for the GaN MEMS resonator (sample A) is *in situ* measured in the vacuum chamber as a function of time. The length of the GaN MEMS resonator is 150 μm with a width of 8 μm. Figure 6(a) shows the shift of the resonant frequency dependent on the time and the extracted mass variation with time is shown in Figure 6(b). With the *in situ* measurement and accurate estimation of the resonant frequency, a high resolution of 10^−12^ g/s is obtained. It is also noted that higher resonant frequencies lead to higher mass resolution, which indicates the GaN MEMS resonator with a large tensile stress is more attractive for the mass sensing.

## 4. Conclusions

In summary, we demonstrated that a large tensile stress simultaneously enhances the resonant frequency and greatly improves the *Q*-factor for the GaN double-clamped MEMS resonator fabricated from the GaN-on-Si system. The ultra-high *Q*-factor exceeding 10^5^ and 6.6-fold enhanced resonant frequency at the tensile stress of 640 MPa are demonstrated. The enhancement of *f* × *Q* product leads to a high resolution of 10^−12^ g/s for the GaN MEMS resonator as the mass sensor for the microdroplet evaporation process. An accurate evaluation of the internal residual stress is developed from the resonant frequency measurement, which shows much higher resolution compared to the Raman spectra. This work is beneficial for the development of highly sensitive and reliable wide-bandgap semiconductor MEMS sensor devices.

See the *supplementary material* for resonant frequency property, Raman spectra, and finite element simulation of sample C.

## Supplementary Material

Supplemental Material
