# Supplementary material for: Strain-enhanced high Q-factor GaN micro-electromechanical resonator
Source: Sci Technol Adv Mater. 2020 Jul 27;21(1):515–23. doi: 10.1080/14686996.2020.1792257 (PMC7476523; doi:10.1080/14686996.2020.1792257)
Supplement: Supplemental Material [file TSTA_A_1792257_SM1397.docx]

*Supplementary Information (Science and Technology of Advanced Materials)*

Strain-enhanced high *Q*-factor GaN micro-electromechanical resonator

Liwen Sang,^a,d*^ Meiyong Liao,^c^ Xuelin Yang,^b^ Huanying Sun,^a^ Jie Zhang,^b^ Masatomo Sumiya,^c^ and Bo Shen^b^

^a^ International Center for Materials Nanoarchitectonics (MANA), National Institute for Materials Science (NIMS), 1-1 Namiki, Tsukuba, Ibaraki 305-0044, Japan; ^b^ Amano-Koide Collaborative Research Lab, National Institute for Materials Science, (NIMS), 1-1 Namiki, Tsukuba, Ibaraki 305-0044, Japan; ^c^ Wide Bandgap Materials Group, National Institute for Materials Science (NIMS), 1-1 Namiki, Tsukuba, Ibaraki 305-0044, Japan; ^d^ State Key Laboratory of Artificial Microstructure and Mesoscopic Physics, School of Physics, Peking University, Beijing 100871, China

^*^ Corresponding and requests for materials should be addressed to Dr. Liwen Sang (email: [SANG.Liwen@nims.go.jp](mailto:SANG.Liwen@nims.go.jp))

In this supplementary material, we provide the information of the MEMS resonators fabricated from sample C with the 2-μm thick GaN on Si substrate.

Figures S1 are the resonance frequencies dependent on the length of the double-clamed bridge. As can be seen, the resonance frequency scales well with the *1/L*, which is similar to sample A and B. This indicates that the strain inside the beam plays an important role in the GaN bridge resonators.


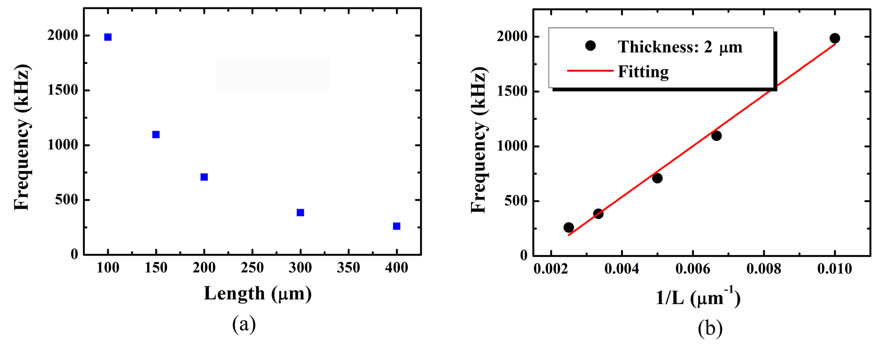


Figure S1 The characteristics of the resonance frequencies *vs* bridge length for sample C.

Figure S2 shows the Raman spectra for the resonator with the 200 μm-length before and after releasing Si.


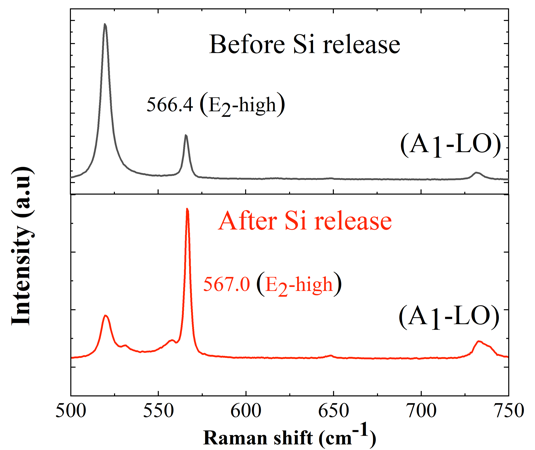


Figure S2 Raman spectra for the resonator before and after releasing Si.

The resonance mode and the eigenfrequency of sample C from the finite element simulation is shown in Fig. S3.


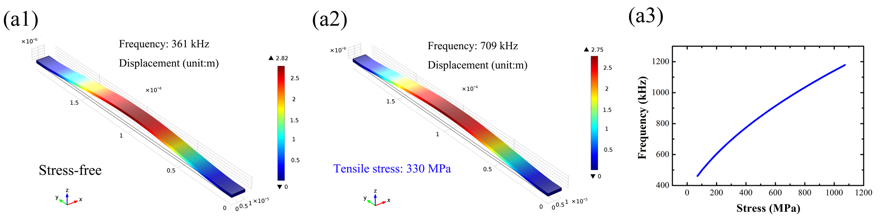


Figure S3 The finite element simulation for sample C in the length of 200 μm (a1) without stress and (a2) with tensile stress of 330 MPa, and (a3) is the dependence of the resonance frequency on the internal stress.
